# Molecular detection and characterisation of herpesviruses in asymptomatic Russian sturgeon (*Acipenser gueldenstaedtii*) from European aquaculture

**DOI:** 10.2478/jvetres-2025-0028

**Published:** 2025-05-21

**Authors:** Sven Michael Bergmann, Matthias Todte, Lea Jäger, Fermin Georgio Lorenzen-Schmidt, Yeonwha Jin, Sandro Klafack, Matthias Lenk, Dewi Syahidah, Bernadetta Rina Hastilestari, Tanjung Penetaseputro, Jean-Christophe Avare, Jeeyoun Hwang, Jolanta Kiełpińska

**Affiliations:** 1 City University of Hong Kong, Jockey Club College of Veterinary Medicine and Life Sciences, Hong Kong; 2 Avicare Plus, 06366 Köthen, Germany; 3 University of Greifswald, 17464 Greifswald, Germany; 4 Friedrich-Loeffler-Institut (FLI) 17493 Insel Riems, Germany; 5 Research Center for Veterinary Science, National Research and Innovation Agency, 16911 Cibinong, Indonesia; 6 Research Institute for Development, University of Montpellier, 34394 Montpellier Cedex, France; 7 National Fishery Products Quality Management Service, 49111 Busan, Republic of Korea; 8 Department of Aquatic Bioengineering and Aquaculture, West Pomeranian University of Technology, 71-550 Szczecin, Poland

**Keywords:** Acipenseridae, virus detection, *in-situ* hybridisation, virus diseases

## Abstract

**Introduction:**

In Germany, around 150,000 kg of mostly Siberian sturgeon (*Acipenser baerii*), were produced in 2021. Sudden mortalities affected negative control Russian sturgeon in experimental infection of several European aquacultured fish species with tilapia lake virus (TiLV). An investigation sought the causative agent. In most of the sturgeon, a specific herpesvirus was detected which also occurred in the carp, crucian carp and tench subjects, but not in Nile tilapia. This herpesvirus was latent in the sturgeon population but became productive to cause the outbreak after three weeks of experimentation.

**Material and Methods:**

Different European aquacultured fishes were experimented upon. Chosen PCRs, nested PCRs and re-amplifications were carried out to identify the causative agent of the mortality event. Sequence analysis of the obtained PCR fragment and *in-situ* hybridisation (ISH) using tissue sections of the experimental fishes were performed.

**Results:**

The PCRs used for detection of *Acipenser* herpesvirus (AciHV)-1 and -2 were always negative. An additional PCR assay with lesser specificity for AciHV found 118 of 123 sturgeon samples and some samples of cohabited cyprinids positive. The similarity of all isolates was 99.7%. The PCR results were confirmed by ISH using probes based on the same sequence, which detected identical viral sequences in both sturgeon and cyprinid samples. These findings revealed that a sterlet sequence previously deposited in the NCBI database had been incorrectly classified.

**Conclusion:**

It seems that different herpesviruses and/or a new subspecies of AciHV are widespread in European farmed sturgeon populations, which, at least for aquacultured fish, opens up the possibility of vaccination against the disease which they cause. Additionally, a more specific diagnostic PCR has to be established.

## Introduction

Sturgeons are taxonomically placed in the order Acipenseriformes and are known to have existed 200 million years ago in the Lower Jurassic (2). Since then they have hardly changed in shape or morphology. They are extremely well adapted to their natural variable environment by evolution. The majority of sturgeon species spend their adult stage as anadromic migratory fish in marine environments or in brackish water before spawning upstream in the big rivers. The sturgeon’s defensive scutes, hydrodynamic shape and fast growth serve first as protection against predators until sexual maturity (1). Male Russian sturgeon (*Acipenser gueldenstaedtii*) will mature in the wild within 8 to 13 years, the females within 10 to 16 years. However, like many other highlyadapted creatures, sturgeons appear to be unable to bear the consequences of human intervention in nature, so that more and more wild populations are dwindling in an alarmingly short time (1). Additionally, the sturgeon is susceptible to infections with a number of viruses. Since the early 1980s, sturgeon farms in California (USA) have recorded increasing numbers of losses of juvenile fish, which were initially unexplained. The causative agents were later identified as white sturgeon adenovirus (12) and white sturgeon iridovirus (10). In 1989, a herpesvirus was isolated that was identified as the cause of increased mortality in Californian sturgeon farms in association with severe infections of the outer layer of the skin (11).

A feature of cypriniviruses and ictaluriviruses, contrary to the usual host specificity of herpes viruses, is the ability to infect other species than their usual respective cyprinid and hosts. This imperfect host accuracy of these viruses can also be related to the severe-to-fatal disease outbreaks and pronounced symptoms that are unusual for herpesviruses (5). Transmission usually occurs from fish to fish by water or through direct contact between infected and uninfected animals. Vaccination being presently impossible, the most successful way to avoid outbreaks is to combine good sanitation, proper diagnosis and identification of the disease’s triggering agents (5). The majority of known data regarding sturgeon herpesviruses is limited to North America; little-to-no data is available from Asia and Europe despite the practice of aquaculture in these regions. Sporadic studies from Russia (19) and Italy (14, 15) suggest that European farms are also infected with different herpesviruses. The herpesviruses detected in Italian aquaculture showed genetic similarities to AciHV-1 (15), while the isolates from the European part of Russia appeared to be related to AciHV-2 (19). Hardly any investigations have been conducted for diagnosis, prevention or vaccination against a possibly latent virus in European and global aquacultured and wild sturgeon. In this study the first step is taken in addressing these needs.

## Material and Methods

### Animal experiment

A total of 123 Russian sturgeon (*A. gueldenstaedtii*) without any clinical signs were cohabited twice for at least 65 days in total with tench (*Tinca tinca*), Nile tilapia (*Oreochromis niloticus*), crucian carp (*Carassius carassius*) and common carp (*Cyprinus carpio*) for investigation of propensity to infection with tilapia lake virus. This TiLV experiment (authorised by veterinary authorities and animal welfare committee under number 7221.3-2-025/19, FLI 23/19) was considered to have no influence on the disease of sturgeon subsequently taken under investigation. Individual specimens of the fish species cohabited with the sturgeon were also tested for acipenserid herpesvirus in random samples. Sturgeon started to die after 30 days of the experiment in both infected groups and contact-free uninfected controls.

### DNA/RNA extraction and PCR selection

At the start it was not known if the agent infecting sturgeon lethally contained DNA or RNA. Therefore, both nucleic acids were extracted. The primer pairs used are presented in Table 1.

**Table 1.** Primer pairs used in this study for DNA and RNA extraction from Russian sturgeon in a mortality event and from cohabited tench, Nile tilapia, crucian carp and common carp

Name	Sequence	Reference
Stör-HV-1F	5′-CGG AAT TCT AGA *Y*TT *Y*GC *IWS IY*T *I*TA *Y*CC-3′	(9)
Stör-HV-1R	5′-CCC GAA TTC AGA TCT C*I*G T*R*T C*I*C C*R*T A-3′	
Stör-HV-Fn	5′-GCA GCA GAC TAC GTG GTG TAC G-3′	(9)
Stör-HV-Rn	5′-AGT TGG *R*GC AGA TCT TCA TTT CGG-3′	
Terminase-AciHV-1 F	5′-ACC TCG TGT TGA TCG-3′	(15)
Terminase-AciHV-1 R	5′-TCA AAA CTT CCG GGT-3′	
Term F2	5′-GC*M M*G*R* GGA CAG A*W*C CC*M* G-3′	(15)
Termsal-3 Rdeg	5′-GGT GCA CAC *R*CC *M*A*D I*GA CG-3′	
TermExon 1F	5′-CAG GT*B* GA*R* CT*M* ATG *M*G*R* GGG TTT TT-3′	personal
TermExon 1R	5′-CAT *R*AT *KKY D*GT *Y*TT *V*CC *R*CA *Y*TG TC-3′	communication

DNA was extracted from samples with a QIAamp DNA-Mini Kit and RNA with a QIAamp Viral RNA Mini Kit (both Qiagen, Hilden, Germany) according to the manufacturer’s instructions. All samples were processed twice with both kits. This supported the assumption that the infectious agent was one containing DNA, and therefore only DNA was subsequently measured. This was carried out with a NanoDrop spectrophotometer (Thermo Fisher Scientific, Wilmington, DE, USA).

For PCRs and nested PCR, a Go Taq Flexi kit (Promega, Madison, WI, USA) was used according to the recommendations of the manufacturer. The composition of the master mix for all first-round PCRs is shown in Table 2. The thermal profile for the reaction with the Stör-HV-F and Stör-HV-R primers as well as for the nested PCR using Stör-HV-Fn and Stör-HV-Rn was an initial step at 95°C for 2 min and 35 cycles at 94°C for 15 sec, 60°C for 30 sec and 72°C for 1 min. The PCR was finalised with 72°C for 10 min and adjusted to 4°C for overnight runs.

**Table 2.** Preparation of master mixes for first-round PCRs using a 5 μL template to amplify DNA of an aetiological agent infecting Russian sturgeon lethally

Components	volume × 1 (in μL)
Diethyl pyrocarbonate–treated water	11.7
5× Green GoTaq Flexi Puffer (Promega)	5
MgCl2 25 mM (Promega)	2.5
dNTPs 10 mM (Thermo Fisher)	0.25
GoTaq DNA polymerase (Promega)	0.125
Primer premix (1:1) 400 nmol	0.5

Five μL of extracted DNA was used as template for the PCR, and 1 μL was allocated for nested PCRs and for re-amplification if necessary. In the latter case, the water content was increased to 25 μL. All other PCRs with the primer pairs in Table 1 proceeded with an initial step at 95°C for 10 min, followed by 35 cycles at 95°C, 55°C and 72°C for 1 min each and were finalised with a step at 72°C for 7 min. All PCR results were visualised with 1.8% agarose gels containing 1% ethidium bromide and running for 60 min at 60 V according to Reinhard (18), and the size of the amplified fragments was assessed with a DNA ladder (Invitrogen, Carlsbad, CA, USA).

### Purification of amplicons

Signals from PCRs which were positive after nested PCR or re-amplification were cut from the agarose gels under UV light and purified using a QIAquick Gel Extraction Kit (Qiagen) according to the manufacturer’s instructions.

### Sequence analysis

The purified DNA isolates were analysed using an ABI 3500 Genetic Analyzer (Applied Biosystems, Foster City, CA, USA) at the Friedrich-Loeffler-Institut (Insel Riems, Germany). All sequences were identified with the National Center of Biotechnology Information (NCBI) database using the basic local alignment search tool NucleotideBLAST Blastn program.

### *In-situ* hybridisation (ISH)

The ISH procedure was as described by Bergmann *et al*. (3) with minor changes. Probe production was carried out using the PCR conditions for Stör-HV-F/R or Stör-HV-Fn/Rn as appropriate. As an unspecific hybridisation control, a koi herpesvirus (KHV) probe with the primer pair KHV 1Fn/1Rn was applied.

## Results

### Animal experiment

**Fig. 1.**
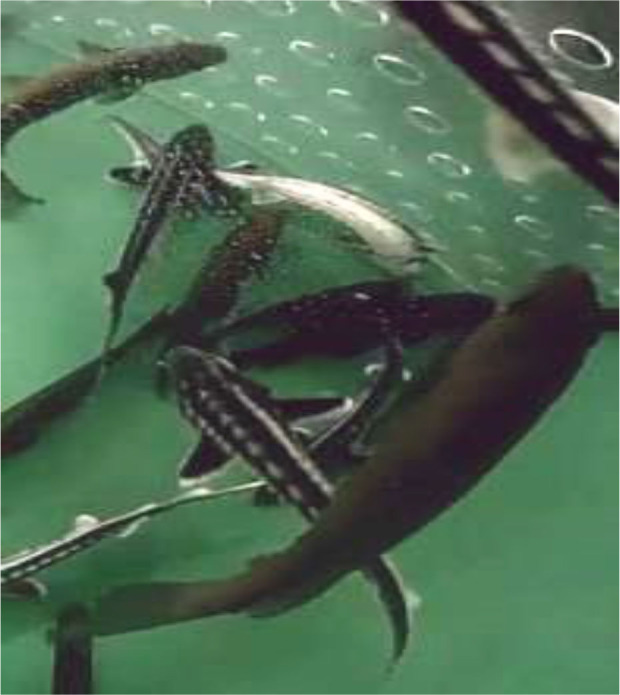
Dead sturgeon lying on the bottom of the tank with bent bodies

When the TiLV experiment had been running for 30 days, an approximately 10 to 15% mortality suddenly occurred in TiLV-infected sturgeon and in the negative control uninfected sturgeon, the latter being housed in another room and maintained by different people. Only some weak petechiae around the fins and the ventral part were visible in fish, both infected and control group individuals. No other clinical signs were observed. In all cases dead fish were lying at the bottom of the tanks with bent bodies (Fig. 1). All dead fish were sampled and frozen at -20°C until further use. Video was recorded of the TiLV experiment over 75 days; however, it brought no additional information regarding the mor tality event in sturgeon. No other fish were affected.

### Extraction of DNA / RNA from fish samples

Both RNA and DNA extraction kits were used with the same samples twice. The extraction kits for RNA operate only on this nucleic acid, but may extract the DNA which is inside the RNA. Measured with the NanoDrop spectrophotometer, it was visible that the DNA extraction kit had up 10 times more DNA inside the eluate than the RNA kit had in its eluate (Table 3), which is guaranteed by the manufacturer.

**Table 3.** RNA and DNA extraction kit concentration of DNA from an aetiological agent infecting Russian sturgeon lethally

Sturgeon sample numbers	RNA extraction kit double-stranded DNA in ng/μL*	DNA extraction kit
388	554.90	893.05
544	150.65	1220.65
577	889.55	889.05
608	240.95	2051.75
Average	459.0125	1263.625

* average value of duplicates

Positive PCR signals were observed with the Stör-HV-1F, Stör-HV-1R, Stör-HV-Fn and Stör-HV-Rn primers. Re-amplification with 2 μL of product from the first-round PCR using Stör-HV-1F and Stör-HV-1R was also successful. An attempt was made to identify the target organ for the suspected herpesvirus in five sturgeons. The organs in which the infectious agent was determined are shown in Table 4. Almost no positive signals were observed in other sturgeon organs.

**Table 4.** Investigation of sturgeon organ tropism using PCR for sturgeon herpesvirus

Organ	Sampling				
	1 (n = 359)	2 (n = 360)	3 (n = 361)	4 (n = 362)	5 (n = 363)
Heart	+	+	+	-	+
Liver	+	+	+	-	+
Spleen	-	+	+	-	+
Kidney	-	-	+	-	+
Pancreas	-	+	+	-	+
Swim bladder	-	+	+	-	+

Positive signals were most frequently found in heart and liver tissue and less frequently in pancreata and swim bladders. Only in two kidneys were positive signals found.

In total 120 sturgeon were sampled and additionally 5 tench, 6 crucian carp, 4 Nile tilapia and 4 common carp. The positive results in PCRs are summarised in Table 5.

**Table 5.** Positive sturgeon herpesvirus PCRs in different fish samples

Species	Positive cases/total	Rate (%)	Remarks
Sturgeon	105/120	87.5	Infected
Tench	2/5	40	Infected
Crucian carp	4/6	66.6	Infected
Common carp	2/6	33.3	Suspicious but positive
Tilapia	0/4	-	Negative

### Sequence analysis of PCR products from samples from different fish

The PCR products of eight sturgeon, two crucian carp, one common carp and one tench were chosen for sequence analysis. The results were compared with the NCBI GenBank database. All resulted sequence data of the four species had a similarity of 99.7%. According to NCBI database matches with accessions up to 2023, all the obtained sequences were identified as *Acipenser ruthenus* genome assembly, chromosome 48, accession No. OV754656.1. The detected sequences were, however, very similar to that of *AciHV-3* published by Clouthier *et al*. (6) in 2023 (Table 6). Using the results from sequence analysis, all positive fish samples contained parts of the *AciHV-3-like* genome; these parts were not of the sturgeon genome, and specifically were not chromosome 48 of the sterlet.

**Table 6.** Assessments of percentage sequence similarities according to GenBank for PCR products of amplification of a disease aetiological agent isolated from different fishes

Fish	*Acipenser ruthenus*	AciHV-3	Remarks
Common carp	96%	99%	
Tench	97%	99%	
Crucian carp 1	97%	99%	AciHV-3-like
Crucian carp 2	95%	96%	
Sturgeon 1	97%	99%	
Sturgeon 2	90%	92%	Suspicious
Sturgeon 3	96%	98%	
Sturgeon 4	97%	99%	
Sturgeon 5	97%	99%	AciHV-3-like
Sturgeon 6	96%	98%	
Sturgeon 7	97%	99%	
Sturgeon 8	97%	99%	
Average value	96%	98%	
Median value	97%	99%	

### Confirmation of sequence analysis results by ISH

**Fig. 2.**
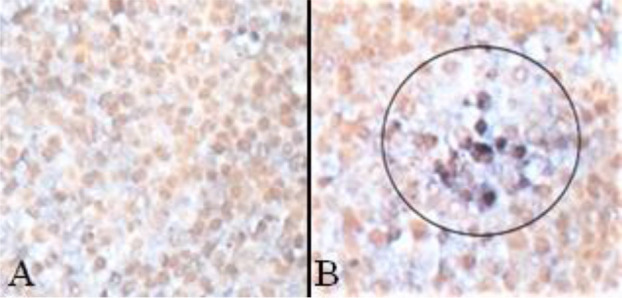
Detection of an *AciHV-like* virus in spleen tissue. A – irregular KHV probe; B – *AciHV-HV* probe

**Fig. 3.**
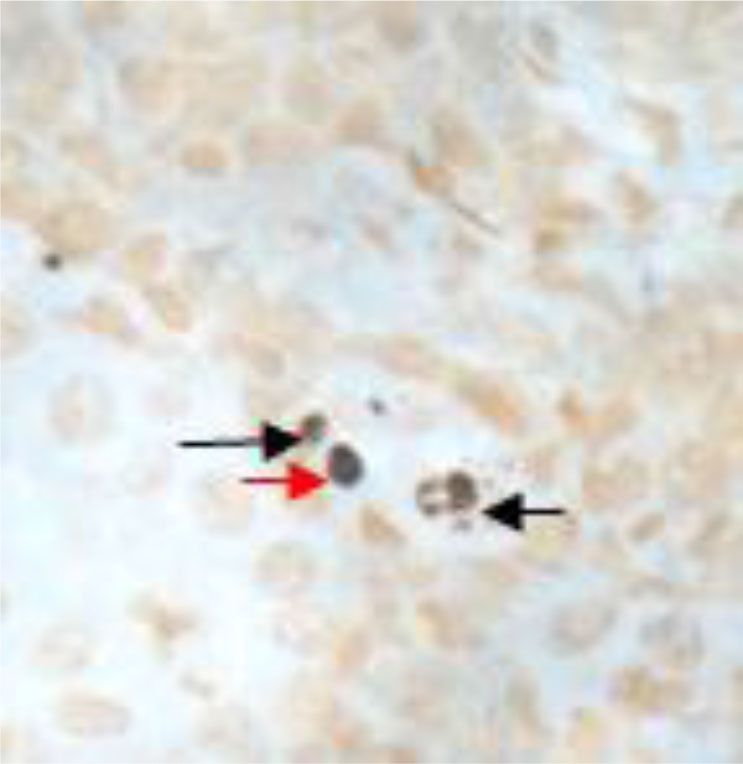
AciHV-like signals in kidney tissues. Red arrow – positive signals; black arrows – melanomacrophages

To confirm that the amplicon was not a genomic part of the animal, ISH was carried out. While all controls including the irregular KHV DNA probe showed no specific labelling of cells, an often single-cell positive reaction was found in different organ tissues. Positive signals were blackish-blue or blackish-purple with yellow negative cells (Fig. 2A) in the surrounding tissue and had to be strongly bound to a cell structure. As examples, sturgeon herpesvirus-positive cells were found in the spleen (Fig. 2B) and kidney (Fig. 3). In Fig. 3 the brownish cells are melanomacrophages after BBY counterstaining and the blackish-blue spots are herpesviral DNA bearing cells. The AciHV-3-like virus was also detected in the tissues of common carp and crucian carp (data not shown).

## Discussion

Sturgeon are farmed worldwide. The main manufacturer, with 104,280 t and 84% of the total world production in 2020, is China (8). In the EU Italy, Poland and Bulgaria are the countries with the highest-valued sturgeon production (8), and the fastest increase is being achieved in Poland. Caviar is produced mainly in China, Italy, Poland and Germany (7). Beside caviar, sturgeon meat is also becoming more and more popular. Almost 100% of sturgeon products presently come from aquacultured fish (8). The danger of accumulating diseases is a given in aquaculture. The highest risk occurs from viral disease, especially from those which may be latent infections. These diseases can be infections and clinical outbreaks caused by different herpesviruses (15), but can also be infections and outbreaks for which iridoviruses are responsible (16). Both virus groups can remain undetected in apparently healthy sturgeon without any clinical signs. Those fish can transfer the viruses after transportation or other stresses. Then sudden, initially inexplicable mortalities with and without clinical signs may occur. The main focus needs to be on the diagnostic techniques to find those viruses or other disease agents (17). In this study a mortality event occurred in Russian sturgeon obtained from a hatchery in Poland. Those fish were used for TiLV trials, during which clinical signs appeared in noninfected controls as well as in TiLV-infected groups. Initial investigations were undertaken for the detection of iridoviruses and herpesviruses, which are the main disease agents in sturgeon aquaculture (13). Originally, RNA or DNA viruses were assumed, but the study quickly became focused on herpesviruses, because firstly, routine bacterial examinations showed no connection to the mortality events, and secondly, the clinical signs in the skin of affected fish were only minimally visible.

Only one PCR, including nested PCR and reamplification PCR, was positive. After sequence analysis, strong identity between isolate sequences and the sequence of the post-2023 entry under accession No. OR242755.1 (sturgeon herpesvirus) and the sequence of the pre-2023 entry under accession No. OV754656.1 (sterlet genomic sequence) were visible in GenBank. The fish genomic origin of the sequence was excluded by ISH using a specific DNA probe to detect sturgeon herpesvirus. If the found sequence had been a fish genomic part, all cells in the tissue would have been labelled. In samples from sturgeon and other fish, it was shown that only some cells with tissue and cell connections were tagged as positive by ISH. It can be assumed that the sterlet genome assembly of chromosome 48, which was directly logged by the Sanger Institute (Hinxton, UK) in 2022, contained a genomic part of a herpesvirus and had been falsely interpreted. Fortunately, in 2023 a new herpesvirus was detected in sturgeon in the USA. The sequence was more than 99% identical to the sequence found in this study.

Additionally, as described by Bergmann *et al*. (3), it was shown that this sturgeon herpesvirus was not species-specific for the infection but for the disease. No other fish had shown clinical signs connected to this sturgeon herpesvirus named “AciHV-3-like”. More intensive investigations are required, *e.g*. cell cultivation of the virus, application of diagnostic tools to detect the virus at low concentrations, and elucidation of the host range of the infection as well as its routes of transmission of the virus to other fish and back to sturgeon. It is unclear if the detected AciHV-3-like herpesvirus can also infect other sturgeon species than Russian sturgeon.

## Conclusion

It seems that herpesviruses are widespread in sturgeon populations, which at least for aquacultured fish, opens up the possibility of vaccination against the disease. Additionally, a more specific diagnostic PCR has to be established.
